# Mental disorders and criminal legal involvement: Evidence from a national diagnostic epidemiological survey

**DOI:** 10.1371/journal.pmen.0000257

**Published:** 2025-04-09

**Authors:** Jeffrey W. Swanson, Madeline Stenger, Michele M. Easter, Natalie Bareis, Lydia Chwastiak, Lisa B. Dixon, Mark J. Edlund, Scott Graupensperger, Heidi Guyer, Maria Monroe-DeVita, Mark Olfson, T. Scott Stroup, Katherine S. Winans, Marvin S. Swartz

**Affiliations:** 1 Department of Psychiatry and Behavioral Sciences, Duke University School of Medicine, Durham, North Carolina, United States of America; 2 Department of Psychiatry, Columbia University Irving Medical Center and New York State Psychiatric Institute, New York, New York, United States of America; 3 Department of Psychiatry and Behavioral Sciences, University of Washington School of Medicine, Seattle, Washington, United States of America; 4 RTI International, Research Triangle Park, Durham, North Carolina, United States of America; Universidad del Valle, COLOMBIA

## Abstract

Large numbers of adults with mental disorders in the United States are incarcerated or otherwise involved with the criminal legal system. Evidence is lacking on prevalence of specific psychiatric diagnoses in this population. This article presents results from the Mental and Substance Use Disorders Prevalence Study (MDPS), a national epidemiological survey that assessed lifetime prevalence of schizophrenia-spectrum disorder, and past-year prevalence of bipolar 1, obsessive-compulsive, major depressive, generalized anxiety, and post-traumatic stress disorders using the Structured Clinical Interview for DSM-5 (SCID 5). Diagnoses and demographic characteristics are compared among three populations: (1) imprisoned adults (N=321), (2) adults not incarcerated but with justice involvement in the past year (N=269), and (3) adults with no criminal legal history in the past year (N=5,004). The weighted sample included households, prisons, hospitals, and homeless shelters. About 4 in 10 people with any past-year criminal legal involvement had at least one of these mental disorders. The cumulative prevalence of these disorders was highest among those in prison (41.8%; 95% Confidence Interval (CI): 33.1-50.9%), lower in the community resident population with criminal legal involvement (37.0%; 95% CI: 25.1-50.6%), and lowest among those with no criminal legal involvement in the past year (24.4%; 95% CI: 21.9-27.0%). Findings for schizophrenia-spectrum disorder followed this pattern: prevalence in prison was 6.3% (95% CI 2.7-14.0%), while among community residents with criminal legal involvement prevalence was 4.4% (95% CI: 1.9-10.0%), and among those with no criminal legal involvement it was 1.7% (95% CI: 1.2-2.4%).

In the latter half of the 20^th^ century, a confluence of two policy-driven trends – the emptying of large public mental hospitals throughout the United States and the rise of mass incarceration – redefined the nation’s response to people with serious mental illnesses like schizophrenia and fundamentally altered the conditions in which they would live [1]. The forces that drove deinstitutionalization and fueled the modern “prison industrial complex” [2] overlapped but were different, as were the populations they most affected; there was no simple substitution of jails and prisons for asylums [3]. Still, large numbers of former, current, and would-be psychiatric patients each year are arrested and booked into jails, are sentenced to prison, or released under community correction supervision [4]. These populations vary in their psychiatric symptoms and degree of impairment as well as criminogenic characteristics and needs. We still do not know enough about them to effectively address the range of social and clinical problems they represent.

Evidence is lacking on the national prevalence of specific psychiatric diagnoses and associated demographic descriptors of people who are incarcerated or living in the community while involved with the criminal legal system. Such evidence is necessary to guide federal and state policies to meet the complex needs of populations at the intersection of behavioral health and criminal justice. To that end, this article presents results of a new national epidemiological survey, the Mental and Substance Use Disorders Prevalence Study (MDPS) [5], which enables a comparison of the prevalence of 6 psychiatric disorders and the demographic characteristics of people with mental illnesses in prison (PR), those who are not incarcerated – mostly residing in the community, though a small number were hospitalized – who had criminal legal involvement in the past year (CCL), and those with no criminal legal history in the past year (No CL). We also provide an estimate of the proportion of people with mental disorders and criminal legal involvement who received any mental health treatment in the past year.

## Background

In 1970, nearly 400,000 adults with psychiatric illnesses were housed in state and county mental hospitals [6], while approximately 360,000 adults were incarcerated in prisons and jails in the United States [7]. Four decades later, by 2010, these numbers had moved dramatically in opposite directions; the census of public mental hospitals had dwindled to under 50,000 while the incarcerated population had risen steeply to more than 1.6 million on any given day [8]. Evidence emerged that these trends could be intertwined; 44% of jail inmates and 37% of federal and state prisoners reported that a mental health professional had told them they have a mental disorder, according to a national survey of inmates published by the Bureau of Justice Statistics (BJS) [9].

Beginning around 2010, criminal justice policy reforms and other macrosocial factors led to a gradual decline in incarceration in the United States; the overall rate of imprisonment per 100,000 population had fallen nearly 10 percent by 2016. However, during the same roughly 5-year period, the proportion of the imprisoned population estimated to have mental illness rose by 11%, as seen in a comparison of two waves of the BJS National Inmate Survey [10]. Today, far more adults with disabling psychiatric illnesses were to be found in jails, prisons, homeless shelters or living on the streets than ever resided in the largest mental hospitals before deinstitutionalization began in the late 1950s [3,11,12].

In the parlance of some social scientists, the “criminalization of mental illness” essentially “transinstitutionalized” [13] psychiatric patients from hospitals to jails and prisons. However, a vigorous debate has persisted over the extent to which the shrinkage of public mental hospital capacity, coupled with the withholding of adequate resources for community-based care, is directly or indirectly responsible for pushing people with serious psychiatric disorders into the criminal legal system [14]. Several driving factors in the 20^th^ century were implicated in the devolution of long-term, hospital-based, and largely custodial care of adults with mental illness [15]. There was the rising cost to states of maintaining enormous treatment facilities with thousands of beds, along with Medicaid financing of short-term general hospital inpatient psychiatric stays that allowed states to transfer substantial costs to the federal government; the advent of more effective pharmacotherapies for controlling the symptoms of mood and psychotic disorders; the impact of scholars’ ethical critiques of the asylum as an instrument of social control [16,17]; and the civil-rights-animated reforms of involuntary commitment laws [18] -- making it difficult to confine non-dangerous patients who simply needed treatment -- and the hopeful advocacy for salubrious community-based models of care [15].

At the same time, powerful countervailing forces undermined the therapeutic mission of deinstitutionalization, setting the stage for the criminal justice system to contend with the deficiencies and challenges created from this shift [19]. On the political front, federal legislation, such as the Omnibus Budget Reconciliation Act of 1981, effectively defunded public community mental health centers in the 1980s [19,20]. The supply of low-cost housing in large urban centers dwindled. The increasing availability of illegal drugs created conditions for substance dependence in many vulnerable individuals with underlying psychiatric disorders, exacerbating their symptoms, and making it difficult for them to follow recommended regimens of psychotropic medication, which in turn led to more frequent relapses, crises, police encounters, and drug-related arrests and criminal charges. The nation’s “war on drugs” and determinant sentencing laws combined with these other social determinants to create conditions for the ensuing large-scale incarceration of people with serious mental illnesses [21,22].

For a variety of reasons, then, by the end of the 20th century a large, institutionalized criminal legal system and infrastructure had emerged in the United States in which a substantial proportion of adults with serious mental illnesses became involved following deinstitutionalization; that carceral system largely remains today as a functional crisis destination for many people with persistent, disabling psychiatric disorders. The mere fact that so many people with mental illnesses are involved with the criminal legal system resonates with and tends to reinforce the public’s belief that serious mental illness causes violence. Empirical research paints a different, and more complex picture. A Connecticut study of criminal justice involvement in a population of more than 25,000 adults diagnosed with schizophrenia or bipolar disorder found that only 10% of arrests were for violent crimes; most were for miscellaneous minor offenses [23]. The Connecticut study also revealed a complex pattern of criminal legal involvement, ranging from a single misdemeanor arrest to lengthy incarceration in a state prison. Most commonly, justice-involved people with mental illness received mental health treatment in different settings at different times, delivered both from the behavioral health system and the criminal justice system. Some individuals in the Connecticut study experienced admissions both to a psychiatric hospital and a jail or prison within the study period, even multiple times, with intervening periods spent in the community.

Addressing the complex causes of this problem – that so many people with mental illnesses are bound up in the criminal legal system – requires understanding not only the problem’s beginnings and historical arc, but its unique features and reach today. How prevalent are different psychiatric diagnoses in these populations, and what are the common social-demographic characteristics of the people who have them?

Numerous studies have attempted to count and describe people with mental illness in the criminal legal system in the United States, and meta-analytic reviews have lined up the existing prevalence studies. Estimates vary considerably, depending on sample setting, methods and measures. The BJS National Inmate Survey (NIS) found that 14% of federal and state prisoners and 26% of jail inmates reported experiences that met the threshold for serious psychological distress in the past 30 days [9]. Within the NIS, serious psychological distress was defined as receiving a score of 13 or more on the Kessler 6 (K6) scale. The K6 is a short, widely used, non-specific psychological distress scale that screens participants for serious mental illness based on how often individuals experience symptoms of psychological distress (e.g., hopelessness, worthlessness) [24]. The NIS used the K6 as a proxy measure for serious mental illness. The most recent National Survey on Drug Use and Health (NSDUH) estimated that mental illness, defined generally, affects approximately 35 percent of community-dwelling adults with a self-reported recent history of criminal legal involvement [25]. A systematic review by Prins [26] described wide variations in prevalence estimates in prisons, noting that many studies utilized prison health records without any independent diagnostic review. Prevalence estimates for major depression ranged from 9% to 29%, and for bipolar disorder from 5.5% to 16.1% across the studies reviewed. The National Inmate Surveys lumped together with a blunt indicator everyone with any mental disorder or those scoring above a threshold of psychological distress on the K6 instrument [27]. Some studies have assessed specific diagnostic prevalence but without the precision of a trained clinician’s interview. Most are limited to a single state, jurisdiction, or service setting. Some studies that use clinician interviewers have done so among patients confined in highly selective forensic facilities, and thus have limited applicability in broader policy discussions that pertain to mental health services for recipients in the general community.

In summary, while previous studies have been informative in their own ways, all are limited. Threats to validity come from use of varying and/or inadequate methods, lack of sample generalizability, and omission of relevant comparison groups not affected by criminal legal involvement. Very few past studies have employed clinician evaluators with research-validated diagnostic instruments, particularly those able to make reliable diagnoses of psychotic disorders. Psychiatric epidemiologic studies in justice populations have also rarely included persons in community corrections under parole or probation. What has been lacking is a national picture of the psychiatric diagnostic and demographic profile of justice-involved adults in different settings.

### The current study

In this study, we report prevalence estimates for non-elderly adults in recent contact with the criminal legal system, or in prison, from the recently completed SAMHSA-sponsored MDPS, which conducted a clinician administered Structured Clinical Interview for DSM-5 Disorders (SCID-5-NSMH [National Study of Mental Health]) [28]. The MDPS included a national household sample as well as a nationally representative prison sample. We also compare prevalence estimates among individuals reporting criminal legal involvement in the past 12 months living in the community (i.e., households, psychiatric hospitals and shelters). Because assessment of substance use disorder was considered too sensitive for a prison sample, these disorders were not assessed in the prison sample, and are not reported here. However, the high prevalence of substance use disorders among incarcerated individuals is well established from other research studies [29].

### Ethics statement

MDPS protocols, instruments, and consent forms were reviewed and approved by the Advarra Institutional Review Board (Pro00042170). RTI and all partner sites entered into reliance agreements with Advarra. Informed consent was obtained prior to the start of each interview. The study had a waiver of the requirement for signature by the participant; documentation of consent was signed by the interviewer.

### Data source and sample

Data for this study were obtained from semi-structured clinical interviews. Data collection occurred between October 26, 2020 and October 22, 2022. All clinical interviewers had appropriate educational backgrounds (e.g., graduate students in American Psychological Association-accredited clinical and counseling psychology PhD programs, masters’‐level social workers, and masters’‐level researchers in mental health or a related field); participated in rigorous training activities; and were required to pass a certification process for NetSCID, a computerized version of the SCID-5-NSMH. To ensure interview quality, individuals possessing doctoral degrees in psychology or social work were hired as clinical supervisors. Quality control processes were performed throughout data collection. For instance, video recordings of ten percent of all completed interviews were evaluated using a standardized scoresheet and inter-rater reliability was monitored through quarterly exercises relying on scoresheet reviews to assess and implement calibration or retraining as needed to ensure consistency across clinical interviewers [30].

A complex sampling design was implemented to obtain robust data from both household and non-household settings (i.e., prisons, state psychiatric hospitals, and homeless shelters). A probability-based design was used for the household sample. The multi-stage design used for residential households included: a roster to identify eligible household residents (25,752 completed out of 234,270 households; weighted response rate (RR) 17.4%); a screener to assess respondent’s level of risk for mental disorders (29,084 individuals completed out of 41,868 invited individuals; weighted RR 67.4%); and clinical interview (4,764 respondents out of 12,906 individuals invited; weighted RR 31.2%). This multi-stage process resulted in an overall weighted response rate of 3.7% for the household sample.

A screening interview was not conducted among the non-household samples due to the anticipated high rates of schizophrenia spectrum disorders and other disorders among these samples compared to the household sample. The nationally representative sample of state and federal prisons included fifty prisons randomly selected from a national list provided by the Bureau of Justice Statistics, of which twenty-two agreed to participate. Participating prisons provided a roster of current individuals who were study eligible (i.e., aged 18–65 years) and included key characteristics (e.g., age, time of admission). The roster was sorted by these characteristics with a randomized sampling scheme to draw a sample based on this sorting. Of the 606 incarcerated individuals sampled from these responding institutions, 321 people completed the clinical interview (weighted RR 49.6%).

State psychiatric hospital and homeless shelter participants were recruited from convenience samples. Twenty-three homeless shelters located in five states and four state psychiatric hospitals from four states were selected to include a range of settings including urban, suburban and rural locations, as well as the variety of populations served (i.e., shelters for single women, single men, serious mental illness, etc.). Similar to the prison sample, state psychiatric hospitals provided a roster of current individuals eligible for the study; the roster was sorted on key characteristics with a randomized sampling scheme to draw a sample based on this sorting. Participating homeless shelters had the option of providing either a roster of their residents or the number of beds at the shelter. Within smaller shelters, all individuals were invited to participate in the clinical interview while a systematic sample of residents or beds was drawn from larger shelters. In total, 171 individuals from state psychiatric hospitals (weighted RR 26.5%) and 423 individuals (weighted RR 32.2%) who were within the homeless sheltered population completed the clinical interviews. Overall, non-household populations were oversampled compared to the household population. This oversampling was addressed using survey weights.

Survey analysis weights were used to calibrate findings to represent all U.S. adults aged 18–65. For instance, weights for the household sample adjusted for selection probability at various stages (e.g., mental health risk stratification from the screener), nonresponse, and alignment with selected characteristics based on 2019 American Community Survey estimates. Analysis weights also scaled the household and non-household samples to their respective size in the U.S. adult population. As such, the household sample accounted for 99.2% of the MDPS target population and associated estimates, while the weight for the non-household sample accounted for 0.8% (i.e., prison sample constituted 0.6%, the state psychiatric hospitals accounted for 0.02% and the sheltered homeless 0.2%). Guyer, Ringeisen, Dever, Liao, Peytchev, Carr, et al. provide a more technical discussion of the MDPS sampling and weighting [30]. The overall process resulted in a national sample of 5,679 adults aged 18–65.

The MDPS screening and clinical interview data are available for restricted use upon approval through the Inter-university Consortium for Political and Social Research (ICPSR 38953) [31].

### Measurement

The goal of the current analysis was to assess and compare the prevalence of six serious mental disorders among individuals with varying levels of involvement with the criminal legal system. Using the following response patterns, participants were divided into three groups: (1) those currently in prison (PR), (2) those with past year criminal legal involvement residing in the community (CCL), and (3) those with no such involvement (No CL) in the past year. Participants within the community, who were not incarcerated (including those in hospitals and homeless shelters), were asked if they had experienced any of the following events in the past twelve months: been arrested/booked due to law violations (excluding minor traffic violations); were on probation or parole; or had a jail or prison stay. If individuals indicated they had experienced one or more of these events, they were classified as having criminal legal involvement (CCL, n=269; weighted 3.1%; 95% CI [confidence interval] 2.3-4.1%). Participants who did not indicate experiencing any of these interactions with the criminal legal system in the past year were classified as having no criminal legal involvement (No CL, n=5,004; weighted 96.3%; 95% CI: 95.3-97.1%). Finally, individuals within the prison sample comprised the third group for analysis (PR, n=321; weighted 0.6%; 95% CI: 0.4-0.7%). We removed eighty-five individuals (<1% weighted sample; 1.5% unweighted) from our analyses who we were unable to classify due to missing data on this key indicator.

Psychiatric diagnosis of major mental disorders was obtained using a modified version of the SCID-5-NSMH. Trained clinicians conducted diagnostic interviews to assess whether participants met criteria for the following disorders in the past year: major depressive disorder (MDD), generalized anxiety disorder (GAD), bipolar I disorder (BPD), posttraumatic stress disorder (PTSD), and obsessive-compulsive disorder (OCD). Measures were also included to determine if individuals met criteria for schizophrenia spectrum disorders (SSD) at any time during their life. SSD included individuals who met diagnostic criteria for schizophrenia, schizoaffective disorder, or schizophreniform disorder. Participants were also asked whether they had received any type of mental health treatment in the past 12 months (i.e., inpatient, outpatient, and/or prescribed medication). A binary variable was created based on these responses to indicate receipt of mental health treatment in the past year.

Demographic characteristics of participants were collected through self-reported measures of sex at birth, age, and racial-ethnic identity.

### Statistical analysis

We produced weighted population estimates which relied on survey weights created to account for the complex sampling design of the MDPS. Weighted bivariate analyses were conducted to examine differences between groups: individuals with no criminal legal involvement in the past year, individuals with criminal legal involvement in the past year who were not incarcerated, and individuals incarcerated within prison. More specifically, we compared results from Chi-square tests (Χ^2^) using 2 by 2 tables (e.g., individuals with no criminal legal involvement in the past year compared to those in prison). The adjusted Rao-Scott Chi-Square statistic, which accounted for the complex sampling design, is reported along with the associated p-value for each analysis, with a threshold of p≤ 0.05 for statistical significance. All analyses were completed using the survey procedures in SAS 9.4.

## Results

### Demographic characteristics

Table 1 summarizes the sample’s distribution on key demographic characteristics by level of criminal-legal involvement. Individuals with criminal legal involvement in the past year were more likely to be male compared to those with no criminal legal involvement (prison, 93.1%; past-year criminal legal involvement, 70.2%; no past-year criminal-legal involvement, 48.4%; prison vs no past-year criminal legal involvement: X^2^=83.2; p<= 0.0001; prison vs past-year criminal-legal involvement: X^2^=13.3, p<=0.001; past-year criminal legal involvement vs no past-year criminal legal involvement X^2^=9.6; p<=0.01). The distributions by racial-ethnic identity also varied substantially across these groups. The proportion of respondents identifying as white, non-Hispanic versus non-white was significantly higher in the subgroup with no criminal legal involvement than in the prison subgroup (X^2^=32.4; p<= 0.0001) and in the subgroup with criminal legal involvement in the past-year (X^2^=5.7; p<=0.05). Approximately 60.5% of individuals with no criminal legal involvement identified as white non-Hispanic compared to only 44.3% of individuals with community-based criminal legal involvement and 25.4% of incarcerated individuals. Within the prison sample, 33.8% of individuals identified as Hispanic/Latino, followed by 27.8% who identified as black, non-Hispanic. Within the community sample with criminal legal involvement in the past year, 22.9% identify as black, while 21.8% identified as Hispanic/Latino—virtually identical proportions.

**Table 1:** Demographic characteristics by level of criminal-legal involvement.

		In Prison			Any criminal-legal involvement in past year			No criminal-legal involvement in past year		
		N	Weighted percent	(95% CI)	N	Weighted percent	(95% CI)	N	Weighted percent	(95% CI)
Race										
	Hispanic/Latino	56	33.8	(13.0, 63.5)	48	21.8	(12.7, 34.8)	761	18.2	(13.5, 24.1)
	White, not Hispanic	143	25.4	(17.3, 35.6)	141	44.3	(32.1, 57.2)	3,140	60.5	(54.7, 66.1)
	Black, not Hispanic	81	27.8	(15.6, 44.6)	46	22.9	(12.0, 39.3)	558	11.9	(9.3, 15.1)
	Other^1^	39	13.0	(7.0, 22.9)	29	11.0	(4.6, 24.1)	533	9.4	(7.7, 11.4)
Sex										
	Male	211	93.1	(87.0, 96.5)	176	70.2	(57.5, 80.4)	1,952	48.4	(44.3, 52.4)
	Female	110	6.9	(3.5, 13.0)	93	29.8	(19.6, 42.5)	3,052	51.6	(47.6, 55.7)

^1^“Other” race includes: Asian, not Hispanic; American Indian/Alaska Native, not Hispanic; Native Hawaiian Islander/Other Pacific Islander, not Hispanic; Multi-racial, not Hispanic.

Those with criminal legal involvement also tended to be younger than their counterparts who did not have criminal legal involvement (mean age in years: prison 38.5; past year criminal legal involvement 37.5; no past year involvement 41.4).

**Fig 1:**
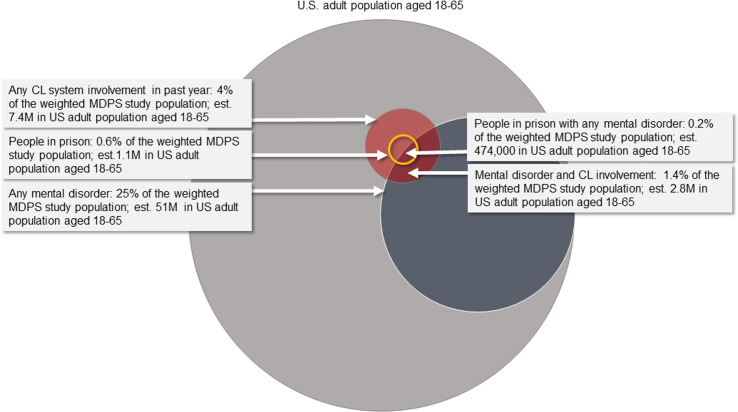
Estimated Prevalence of MDPS mental disorders and criminal legal involvement.

Fig 1 displays the estimated prevalence of any MDPS mental disorder and criminal legal involvement for U.S. adults aged 18–65. Approximately 51 million individuals (25% of the adult population) have at least one of the six mental disorders. An estimated 7.4 million adults (weighted estimate of 3.7%) had contact with the criminal legal system within the past year. This estimate includes 6.2 million individuals with community-based criminal legal involvement (3.1% of the population) and 1.1 million individuals within prison (0.6% of the population). Approximately 2.8 million adults aged 18–65 (weighted estimate of 1.4%) had both a MDPS mental disorder and involvement with the criminal legal system in the past year, including almost half a million individuals incarcerated within prison (0.2% of the MDPS study population). Among those with any criminal legal involvement, approximately 38% had at least one MDPS mental illness. Fig 1 was created using the ‘eulerr’ [32] package in the R programming language [33] and PowerPoint.

### Prevalence of MDPS mental disorders

Table 2 displays the prevalence of MDPS mental disorders by level of criminal legal involvement. Among individuals within prison, approximately 42% of individuals had at least one mental disorder, while 16% had two or more of the disorders examined. The two most common mental disorders among this group were MDD (26%) and GAD (14%). An estimated 9% of individuals within prison met criteria for PTSD, 8% for OCD; 6% for SSD, and 4% for BPD. Among individuals with any past-year criminal legal involvement who resided in the community, approximately 37% had at least one of the mental disorders examined and 13% had two or more of the disorders. The two most common disorders among these individuals were MDD (21%) and PTSD (15%). An estimated 10% of individuals with criminal-legal involvement in the past year met the diagnostic criteria for GAD, 4% for SSD, 3% for OCD, and 1% for BPD. Among those individuals who did not indicate having any criminal-legal involvement in the past year, approximately 24% had at least one of the mental disorders examined and 8% had two or more of the disorders. The two most common disorders among this group were MDD (15%) and GAD (10%). An estimated 4% met criteria for PTSD, 2% for OCD, 2% for SSD and 2% for BPD.

**Table 2:** Prevalence of specific mental disorders by level of criminal-legal involvement.

	In Prison			Any past-year criminal-legal involvement			No past-year criminal-legal involvement		
Diagnosis	N	Weighted Percent	95% CI	N	Weighted Percent	95% CI	N	Weighted Percent	95% CI
Schizophrenia spectrum disorder	21	6.3	(2.7, 14.0)	57	4.4	(1.9, 10.0)	184	1.7	(1.2, 2.4)
Major depression	90	26.1	(18.2, 36.0)	52	21	(13.0, 32.1)	1,239	15.1	(13.1, 17.3)
Generalized anxiety disorder	40	14.1	(6.2, 28.8)	34	10.2	(5.6, 18.1)	864	9.9	(8.1, 12.0)
Bipolar disorder	11	3.6	(1.6, 7.8)	16	1.3	(0.5, 3.1)	133	1.5	(0.9, 2.5)
Post-traumatic stress disorder	33	9.2	(4.0, 19.9)	39	14.9	(7.0, 29.1)	342	3.7	(2.9, 4.6)
Obsessive-compulsive disorder	23	8.4	(2.9, 21.9)	16	3.1	(1.3, 7.0)	218	2.4	(1.7, 3.5)
Any mental disorder	136	41.8	(33.1, 50.9)	147	37	(25.1, 50.6)	1,995	24.4	(21.9, 27.0)
Multiple mental disorders	59	15.7	(6.7, 32.6)	46	13.2	(7.6, 22.0)	756	7.7	(6.6, 9.0)

Table 3 provides the Chi-square test results for comparisons between groups. Differences between individuals in prison and those with past year criminal legal involvement did not reach statistical significance for any of the MDPS disorders examined. In contrast, except for GAD and BPD, differences between those in prison and individuals with no past year criminal legal involvement were statistically significant (SSD Χ^2^=9.3, p<0.01; MDD Χ^2=^8.1, p<0.01; PTSD Χ^2=^4.7, p<0.05; OCD Χ^2=^5.6, p<0.05; Any MDPS mental disorder Χ^2=^17.4, p≤0.0001). Differences in prevalence among individuals with past-year criminal legal involvement and those with no past-year criminal legal involvement were statistically significant for SSD (Χ^2=^4.8, p<0.05); PTSD (Χ^2=^15.1, p≤0.0001); and having any MDPS mental disorder (Χ^2=^4.3, p<0.05).

**Table 3:** Rao-Scott chi-square statistic and p-values for group comparisons on prevalence of mental disorder.

	PR vs No CL		PR vs CCL		CCL vs No CL	
Diagnosis	X^2^	p-value	X^2^	p-value	X^2^	p-value
Schizophrenia spectrum disorder	9.3	0.002	0.4	0.549	4.8	0.028
Major depression	8.1	0.005	0.6	0.437	1.7	0.198
Generalized anxiety disorder	0.7	0.394	0.4	0.516	0.0	0.915
Bipolar disorder	3.4	0.064	3.6	0.059	0.1	0.744
Post-traumatic stress disorder	4.7	0.030	0.8	0.378	15.1	0.000
Obsessive-compulsive disorder	5.6	0.018	2.7	0.102	0.3	0.594
Any mental disorder	17.4	0.000	0.4	0.544	4.3	0.039
Multiple mental disorders	2.8	0.094	0.1	0.721	3.5	0.063

Note: PR, prison; No CL, no criminal legal involvement in the past year; CCL, criminal legal involvement in past year.

Of the 66 individuals residing in the community with an MDPS disorder and any criminal legal involvement in the past year, 31.8% reported receiving no treatment for their mental illness. Treatment included receiving prescription medication, inpatient or outpatient services. By comparison, approximately 40% of those with a mental illness but no criminal legal involvement in the past year did not receive any mental health treatment in the past year.

The imprisoned and not-imprisoned segments of the study population with mental disorder and criminal legal involvement differed in their demographic profiles. Those in the prison sample with mental illness were predominantly male (89.9%) and non-white (73.1%) compared to those who were not in prison (52.6% male; 49.2% non-white). These differences between the incarcerated and community-dwelling segments of the study population with mental illness and criminal legal involvement tended to reflect the characteristics of the general populations in those settings. On average, people in prison tend to be disproportionately male and of minority racial status.

## Discussion

This study offers new evidence from a national psychiatric epidemiological survey to estimate the size, diagnostic composition, and demographic characteristics of the U.S. population of adults with mental illnesses who are involved in the criminal legal system. The study is unique in that it includes both people in prison and community residents with a recent history of arrest, incarceration, or community correction supervision. The study used the clinician-administered SCID-5-NSMH, which provides valid and reliable diagnostic assessments, particularly for schizophrenia spectrum disorders.

We have long known that the downsizing and closure of many state mental hospitals in the decades spanning the 21^st^ century left too many adults with potentially disabling mental health conditions untreated, uncared-for, and often unhoused in American urban communities. We have also known that too many of these same adults were swept into the criminal legal system in the tide of mass incarceration and homelessness in the 1980s and 1990s. This article offers a clear diagnostic and demographic profile of that population, and a comparison of its incarcerated and community-resident segments.

The study confirms that major psychiatric diagnoses are substantially more common among adults who are involved with the criminal legal system in the United States than among those in the general population, and also more common than among prisoners in other high-income countries, as reported in meta-analytic studies [34]. Moreover, the study found that the prevalence of serious disorders such as SSD and depression was lower in the population without recent criminal legal involvement than in those with recent criminal history and those in prison. The study did not find statistically significant differences in the prevalence of specific mental disorders between the two populations with recent criminal history, but it should be noted that the numeric frequency of most disorders, except PTSD, was higher in the prison population than in the community population reporting criminal involvement in the past year. A similar pattern emerges in the prevalence of having multiple mental disorders; the condition of having multiple specific disorders was found to be nearly twice as prevalent in the prison and community samples with criminal legal involvement (15.7 and 13.2%, respectively) than their non-CL involved counterparts (7.7%). This diagnostic complexity poses an additional challenge in treating these largely underserved populations.

The prevalence of schizophrenia spectrum disorder (SSD) was also highest in the prison sample (6.3%), lower in the community resident sample with criminal legal involvement (4.4%), and lowest in the sample without criminal legal involvement (1.7%). Beyond the general phenomenon of the criminalization of mental illness, several additional factors might explain the gradient in the prevalence of schizophrenia across these sample segments. Schizophrenia is an especially serious condition that, if untreated, can cause severe impairment of social functioning. Within the population of adults with any criminal legal involvement, individuals with schizophrenia could be overrepresented in the group that is charged with more serious offenses, who receive more severe sentences and are more likely to serve out their sentences in prison rather than under community supervision. This would be consistent with the reality that many people with schizophrenia currently receive little or no treatment in the community [35], and that untreated psychosis increases the risk for substance use disorder, excessive threat perception, interpersonal conflict, poor social functioning in general, and exposure to stressful and criminogenic environments. Evidence from another analysis relying on the MDPS survey estimated that only 26% of adults with schizophrenia spectrum disorders residing in households received minimally adequate treatment in the past year, which was defined as having four or more outpatient visits for mental health and taking one or more antipsychotic medications in the past year [36]. Moreover, people with schizophrenia who are convicted of crimes could be less likely to receive community supervision as an alternative to prison, perhaps due to stigma and perceived dangerousness, or lack of strong representation by counsel.

Diverging from the pattern seen with other disorders, the diagnosis of posttraumatic stress disorder (PTSD) was found to be more prevalent in the community population with criminal legal involvement than in the prison sample (14.9% and 9.2%, respectively), though it was also least prevalent in the household sample (3.7%). The most likely explanation for the difference in prevalence between the prison and community sample with criminal legal involvement is covariation with gender: the community sample with criminal legal involvement had a higher proportion of women than the prison sample, and women had higher rates of PTSD, as is the case with depression.

People with mental illness in the criminal legal system comprise a heterogeneous group: some of them likely resemble the larger population with mental illness and no criminal involvement, while others resemble the population with criminal legal involvement and no mental illness. Accordingly, service systems and policies to address the needs of these populations must be tailored to individuals with a variety of reasons for their involvement with the criminal legal system, some directly related to psychopathology and others not.

Overall, the persistently high prevalence of mental disorders in the prison and community resident study populations with criminal legal involvement is of concern and suggests a continuing problem with lack of access to effective mental health treatment. Prisoners have a constitutional right to treatment [37], though this does not mean that high quality treatment is available to all prisoners with mental illness. In the community, no such right exists and this population may struggle to access treatment, due to barriers posed by having a mental illness and a criminal record.

Policies such as pre- and post-booking jail diversion, specialty-docket mental health courts, and treatment-focused probation and reentry programs have emerged as part of an effort to address complex problems at the intersection of behavioral health and criminal involvement. But policies to divert people with mental disorders from the criminal legal system cannot work if people are deflected *to* a fragmented and under-resourced public mental health care system that is ill-equipped to meet the array of clinical and social needs of people with compound psychopathologies intertwined with criminal histories.

Our analysis has several important limitations including the reliance on self-reported criminal involvement for individuals who were not within the prison sample; convenience sampling of state psychiatric hospitals and homeless shelters; a low overall-response rate; an inability to disentangle the implications of the COVID-19 pandemic on our results; and the lack of data on substance use disorders. For diagnostic groups with low population prevalence, the frequency counts in the sample cells were quite low, limiting much of the analysis to bivariate comparisons. As these limitations could have influenced the prevalence estimates in either direction, we provided confidence intervals for our estimates.

People with serious psychiatric conditions need appropriate services and treatments; better access to a full continuum of care could prevent many from ending up in the criminal legal system. How to accomplish that goal is a difficult question for policy and research. A reinvestment in community care is needed, especially in some sectors, to replace funding and other resources that disappeared from the budgets of public sector mental health authorities with the advent of privatization and managed care. Packages of intensive, effective, and equitable services that include outreach and access to housing are widely needed [4,38–40]. Expanded use of evidence-based forensic case management models is an appropriate, targeted approach. Studies on Forensic Assertive Community Treatment (FACT) teams provide promising results in reducing recidivism and hospitalizations among individuals involved with the criminal legal system who have severe mental illness. However, further research is needed to better understand the context and circumstances under which such models yield optimal outcomes [41]. In the context of well-resourced community treatment, better-funded outpatient civil commitment programs could help in some cases. Where there are shortages of psychiatric hospital beds and service providers, inpatient treatment capacity should be expanded, allowing sufficient lengths of stay to evaluate treatment response and initiate meaningful recovery. All these solutions require public funding and political will. The present alternative is a humanitarian crisis.

## References

[1] Raphael S, Stoll MA. Assessing the Contribution of the Deinstitutionalization of the Mentally Ill to Growth in the U.S. Incarceration Rate. The Journal of Legal Studies. 2013;42(1):187–222. doi: 10.1086/667773

[2] Schlosser E. The prison-industrial complex. The Atlantic Monthly. 1998;282(6):51–77.

[3] Parsons AE. From asylum to prison: Deinstitutionalization and the rise of mass incarceration after 1945. UNC Press Books; 2018.

[4] Bonfine N, Wilson AB, Munetz MR. Meeting the Needs of Justice-Involved People With Serious Mental Illness Within Community Behavioral Health Systems. Psychiatr Serv. 2020;71(4):355–63. doi: 10.1176/appi.ps.201900453 31795858

[5] Ringeisen H, Edlund M, Guyer H, Geiger P, Stambaugh L, Dever J, et al. Mental and Substance Use Disorders Prevalence Study (MDPS): Findings Report. Research Triangle Park, NC, RTI International. 2023.

[6] Lutterman T. Trends in psychiatric inpatient capacity, United States and each state, 1970 to 2018. National Association of State Mental Health Program Directors Technical Assistance Collaborative Paper. 2022(2).

[7] Cahalan M, Parsons L. Historical corrections statistics in the United States, 1850-1984. Washington (DC): US Department of Justice, Bureau of Justice Statistics; 1986 Dec 1. p. 191.

[8] Ghandnoosh N. Policy Brief: Can We Wait 75 Years to Cut the Prison Population in Half? The Sentencing Project; 2018 [updated 2022 Oct. 18; cited 2024 Nov. 18]. Available from: https://www.sentencingproject.org/policy-brief/can-we-wait-75-years-to-cut-the-prison-population-in-half/.

[9] Bronson J, Berzofsky M. Indicators of mental health problems reported by prisoners and jail inmates, 2011–12. Bureau of Justice Statistics. 2017:1–16.

[10] Maruschak L, Bronson J, Alper M. Indicators of Mental Health Problems Reported by Prisoners: Survey of Prison Inmates, 2016 NCJ252643. Washington (DC): US Department of Justice, Bureau of Justice Statistics. 2021.

[11] Torrey E, Kennard A, Eslinger D, Lamb R, Pavle J. More mentally ill persons are in jails and prisons than hospitals: A survey of the states. Arlington (VA): Treatment Advocacy Center. 2010, p. 1–18.

[12] Lamb HR, Weinberger LE. The shift of psychiatric inpatient care from hospitals to jails and prisons. J Am Acad Psychiatry Law. 2005;33(4):529–34. 16394231

[13] Prins SJ. Does transinstitutionalization explain the overrepresentation of people with serious mental illnesses in the criminal justice system?. Community Ment Health J. 2011;47(6):716–22. doi: 10.1007/s10597-011-9420-y 21655941

[14] Winkler P, Barrett B, McCrone P, Csémy L, Janous̆ková M, Höschl C. Deinstitutionalised patients, homelessness and imprisonment: systematic review. Br J Psychiatry. 2016;208(5):421–8. doi: 10.1192/bjp.bp.114.161943 27143007

[15] Montenegro C, Irarrázaval M, González J, Thomas F, Urrutia J. Moving psychiatric deinstitutionalization forward: A scoping review of barriers and facilitators. Glob Ment Health (Camb). 2023;10:e29. doi: 10.1017/gmh.2023.18 37808271 PMC7615177

[16] Rothman D. The discovery of the asylum: Social order and disorder in the new republic. Routledge; 2017.

[17] Goffman E. Asylums: Essays on the social situation of mental patients and other inmates. AldineTransaction; 1961.

[18] Appelbaum P. Almost a revolution: Mental health law and the limits of change. Oxford (USA): Oxford University Press; 1994.

[19] Yohanna D. Deinstitutionalization of people with mental illness: causes and consequences. Virtual Mentor. 2013;15(10):886–91. doi: 10.1001/virtualmentor.2013.15.10.mhst1-1310 24152782

[20] Grob GN. Public policy and mental illnesses: Jimmy Carter’s Presidential Commission on Mental Health. Milbank Q. 2005;83(3):425–56. doi: 10.1111/j.1468-0009.2005.00408.x 16201999 PMC2690151

[21] Kim D-Y. Psychiatric Deinstitutionalization and Prison Population Growth: A Critical Literature Review and Its Implications. Criminal Justice Policy Review. 2014;27(1):3–21. doi: 10.1177/0887403414547043

[22] Lurigio AJ. People with Serious Mental Illness in the Criminal Justice System: Causes, Consequences, and Correctives. The Prison Journal. 2011;91(3_suppl):66S-86S. doi: 10.1177/0032885511415226

[23] Swanson JW, Frisman LK, Robertson AG, Lin H-J, Trestman RL, Shelton DA, et al. Costs of criminal justice involvement among persons with serious mental illness in connecticut. Psychiatr Serv. 2013;64(7):630–7. doi: 10.1176/appi.ps.002212012 23494058

[24] Kessler RC, Barker PR, Colpe LJ, Epstein JF, Gfroerer JC, Hiripi E, et al. Screening for serious mental illness in the general population. Arch Gen Psychiatry. 2003;60(2):184–9. doi: 10.1001/archpsyc.60.2.184 12578436

[25] Shah H, Hawks L, Walker RJ, Egede LE. Substance Use Disorders, Mental Illness, and Health Care Utilization Among Adults With Recent Criminal Legal Involvement. Psychiatr Serv. 2024;75(3):221–7. doi: 10.1176/appi.ps.20220491 37674397 PMC11451170

[26] Prins SJ. Prevalence of mental illnesses in US State prisons: a systematic review. Psychiatr Serv. 2014;65(7):862–72. doi: 10.1176/appi.ps.201300166 24686574 PMC4182175

[27] Kessler RC, Andrews G, Colpe LJ, Hiripi E, Mroczek DK, Normand SLT, et al. Short screening scales to monitor population prevalences and trends in non-specific psychological distress. Psychol Med. 2002;32(6):959–76. doi: 10.1017/s0033291702006074 12214795

[28] First MB, Williams JB, Karg RS, Spitzer RL. Structured clinical interview for DSM-5 disorders. Clinician Version (SCID-5-CV). Arlington, VA, American Psychiatric Association. 2016.

[29] Maruschak L, Bronson J, Alper M. Alcohol and drug use and treatment reported by prisoners: Survey of prison inmates, 2016. 2021;(Specify report number or identifier here):Specify page range here if applicable.

[30] Guyer H, Ringeisen H, Dever J, Liao D, Peytchev A, Carr C, et al. Mental and Substance Use Disorders Prevalence Study: Background and Methods. Int J Methods Psych Res. 2024;33(1). doi: 10.1002/mpr.2000

[31] Ringeisen H, Edlund M, Guyer H, Dever J, Liao D, Peytchev A. Mental and substance use disorders prevalence study (MDPS), United States, 2020-2022. 2024.

[32] Larsson J. Eulerr: Area-Proportional Euler and Venn Diagrams with Ellipses. R package version 7.0.2; 2024[cited 2025 Jan 2]. Available from: https://CRAN.R-project.org/package=eulerr

[33] R Core Team. R: A Language and Environment for Statistical Computing. R Foundation for Statistical Computing, Vienna, Austria. Version 4.3.2. 2023 Oct 31 [cited 2025 Jan 2]. Available from: https://www.R-project.org/.

[34] Fazel S, Seewald K. Severe mental illness in 33,588 prisoners worldwide: systematic review and meta-regression analysis. Br J Psychiatry. 2012;200(5):364–73. doi: 10.1192/bjp.bp.111.096370 22550330

[35] Mojtabai R, Fochtmann L, Chang S-W, Kotov R, Craig TJ, Bromet E. Unmet need for mental health care in schizophrenia: an overview of literature and new data from a first-admission study. Schizophr Bull. 2009;35(4):679–95. doi: 10.1093/schbul/sbp045 19505994 PMC2696378

[36] Bareis N, Edlund M, Ringeisen H, Guyer H, Dixon LB, Olfson M, et al. Characterizing Schizophrenia Spectrum Disorders: Results of the U.S. Mental and Substance Use Disorders Prevalence Study. Psychiatr Serv. 2025;76(1):2–12. doi: 10.1176/appi.ps.20240138 39308173 PMC11693490

[37] Estelle v. Gamble, 429 US 97, 103. 1976.

[38] Kirkbride JB, Anglin DM, Colman I, Dykxhoorn J, Jones PB, Patalay P, et al. The social determinants of mental health and disorder: evidence, prevention and recommendations. World Psychiatry. 2024;23(1):58–90. doi: 10.1002/wps.21160 38214615 PMC10786006

[39] Shim RS. Dismantling Structural Racism in Psychiatry: A Path to Mental Health Equity. Am J Psychiatry. 2021;178(7):592–8. doi: 10.1176/appi.ajp.2021.21060558 34270343

[40] Watson TM, Benassi PV, Agic B, Maharaj A, Sockalingam S. Community-Based Mental Health and Substance Use Services for People Leaving Prison: Equity and Inclusion Strengths and Limitations in Specialized Service Inventory Development. Community Ment Health J. 2023;59(3):421–7. doi: 10.1007/s10597-022-01050-5 36380033 PMC9667000

[41] Cuddeback GS, Simpson JM, Wu JC. A comprehensive literature review of Forensic Assertive Community Treatment (FACT): Directions for practice, policy and research. International Journal of Mental Health. 2020;49(2):106–27. doi: 10.1080/00207411.2020.1717054

